# Antibody-Mediated Inhibition of Insulin-Degrading Enzyme Improves Insulin Activity in a Diabetic Mouse Model

**DOI:** 10.3389/fimmu.2022.835774

**Published:** 2022-03-08

**Authors:** Ofir Fursht, Mirit Liran, Yuval Nash, Vijay Krishna Medala, Dor Ini, Tabitha Grace Royal, Guy Goldsmith, Limor Nahary, Itai Benhar, Dan Frenkel

**Affiliations:** ^1^ Department of Neurobiology, School of Neurobiology, Biochemistry and Biophysics, the George S. Wise Faculty of Life Sciences, Tel Aviv University, Tel Aviv, Israel; ^2^ The Shmunis School of Biomedicine and Cancer Research, the George S. Wise Faculty of Life Sciences, Tel Aviv University, Tel Aviv, Israel; ^3^ Sagol School of Neuroscience, Tel Aviv University, Tel Aviv, Israel

**Keywords:** antibody, ScFv library, antibody engineering, diabetes, insulin-degrading enzyme (IDE)

## Abstract

Diabetes is a metabolic disease that may lead to different life-threatening complications. While insulin constitutes a beneficial treatment, its use may be limited due to increased degradation and an increase in side effects such as weight gain and hypoglycemia. Small molecule inhibitors to insulin-degrading enzyme (IDE) have been previously suggested as a potential treatment for diabetes through their ability to reduce insulin degradation and thus increase insulin activity. Nevertheless, their tendency to bind to the zinc ion in the catalytic site of IDE may affect other important metalloproteases and limit their clinical use. Here, we describe the isolation of an IDE-specific antibody that specifically inhibits insulin degradation by IDE. Using phage display, we generated a human IDE-specific antibody that binds human and mouse IDE with high affinity and specificity and can differentiate between active IDE to a mutated IDE with reduced catalytic activity in the range of 30 nM. We further assessed the ability of that IDE-inhibiting antibody to improve insulin activity *in vivo* in an STZ-induced diabetes mouse model. Since human antibodies may stimulate the mouse immune response to generate anti-human antibodies, we reformatted our inhibitory antibody to a “reverse chimeric” antibody that maintained the ability to inhibit IDE *in vitro*, but consisted of mouse constant regions, for reduced immunogenicity. We discovered that one intraperitoneal (IP) administration of the IDE-specific antibody in STZ-induced diabetic mice improved insulin activity in an insulin tolerance test (ITT) assay and reduced blood glucose levels. Our results suggest that antibody-mediated inhibition of IDE may be beneficial on improving insulin activity in a diabetic environment.

## Introduction

Diabetes mellitus (DM) is a metabolic disorder that results in chronic hyperglycemia due to insufficient levels or impaired responses to the hormone insulin that is essential for glucose homeostasis (1, 2). The World Health Organization indicates that one in 10 people has diabetes, reaching more than 422 million people worldwide in 2014 (3). The two most common forms of diabetes are due to either a diminished production of insulin (type I diabetes, T1D) or insufficient response by the body to insulin (type II diabetes, T2D) (4). With the aging population growing in both number and percentage, there is an increase in the importance of developing and studying new methods for alleviating diabetes-related complications such as stroke, cognitive diseases, vascular diseases, and kidney and eye diseases (5–8). Those diseases can affect both the quality of life of the patients and their families and add to the burden on the public healthcare system. About 25% of diabetes patients require administration of insulin (9). The majority of the top diabetes drugs are insulin homologues (10). Nevertheless, the problems with insulin treatment are as follows: maintaining the basal levels, due to degradation (11, 12); reluctance of patients to inject insulin continuously due to side effects (weight gain, hypoglycemia) (13); and continued administration of insulin may lead to enhanced clearance by the immune cells (14).

Insulin-degrading enzyme (IDE, insulysin) is a ~110 kDa zinc metalloprotease and is an enzyme that was reported to be responsible for insulin degradation (15). IDE is highly conserved; human and mouse/rat proteins share 95% amino acid identities and 100% identity of the residues located in the putative catalytic site (15, 16). Structural analysis revealed that human IDE consists of two equally sized (~55 kDa) N- and C-terminal domains. Several proteins were shown to be digested by IDE (17); however, insulin has the highest affinity to IDE (K_m_ ≈ 14 nM) (18). The presence of IDE in multiple cellular compartments has been reported (19), including mitochondria, and secreted to the plasma (20, 21). Studies of IDE inhibition (22–24) have shown that modulating IDE activity could potentially be a new therapeutic strategy for treating diabetes (25). We recently reported that IDE levels are elevated in the serum of prediabetes metabolic syndrome patients in correlation with increase in triglycerides and reduction in HDL levels (26). Nevertheless, the problem with some of the IDE inhibitors is their limited specificity, as in addition to inhibiting IDE, they were shown to inhibit other Zn^2+^ metalloproteases (27).

Antibodies are known for their high affinity and specificity for a binding partner (ligand or antigen) (28). The antibody engineering field has evolved rapidly in the past decades, much due to novel technologies for the *in vitro* isolation of antibodies from combinatorial libraries and their functional expression in various systems (29). Antibody-based therapy aims for the production of antibodies that will eliminate or neutralize its target. Thus, in this work, we aimed to develop IDE-specific antibodies and evaluate their potential as a therapeutic approach for DM in a mouse model.

## Materials and Methods

### Expression and Purification of WT and Mutated IDE Proteins

**Figure 1:**
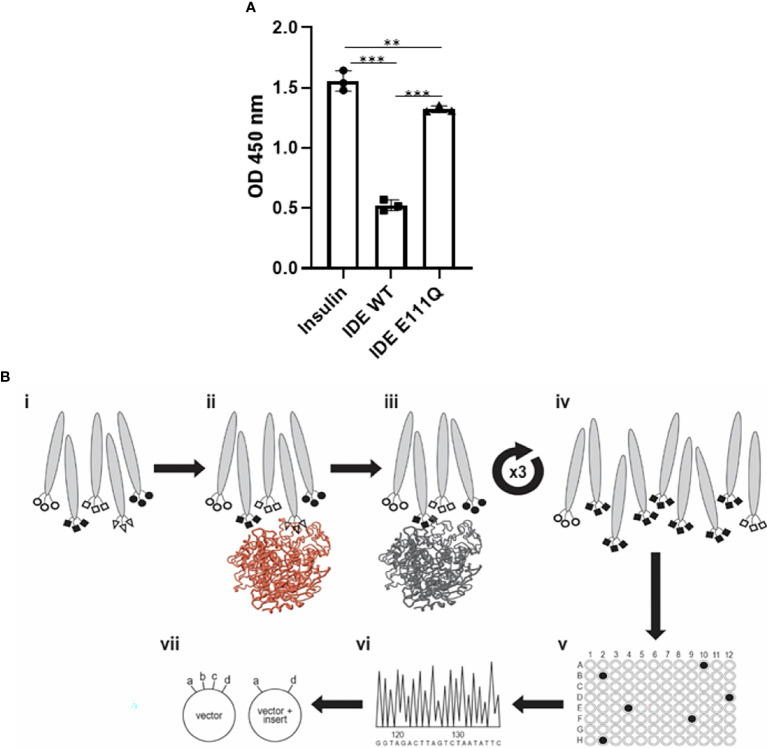
Activity of purified rhIDE wild-type and inactive mutant (E111Q) and their utilization in the antibody discovery process. **(A)** IDE activity assay: incubation of 1.5 µg/L human insulin with PBS (insulin, 1.5 μg/L) or 12 µg/ml rhIDE WT or E111Q for 2 h at 37°C. Residual insulin was later analyzed using Mercodia ultrasensitive mouse insulin ELISA. Results detected on an ELISA plate reader at 450 nm. The results are presented as mean ± SEM (n = 3). **p < 0.01; ***p < 0.001. One-way ANOVA with Bonferroni correction. **(B)** Scheme of the antibody discovery pipeline that was used to obtain rhIDE-binding antibodies: (i) antibody phage display library 2 × 10^9^ antibody clones used as input for the first panning cycle; (ii) depletion: capture on mutant rhIDE (colored brown); (iii) positive selection: capture depleted phages on WT rhIDE; (iv) washed to remove unbound phages, recovered the phages that bound rhIDE, and amplified them by infecting *E. coli* and preparing an enriched population of rhIDE binders to be used as input for the subsequent panning cycles. Repeated three times to obtain a phage population dominated by rhIDE binders; (v) identified phages that bind WT rhIDE by monoclonal phage ELISA; (vi) identified the antibody coding sequences by sequencing of the DNA recovered from monoclonal phages that bind WT rhIDE; (vii) cloned the antibody coding sequences from the phages that bind WT rhIDE to an antibody expression vector to produce soluble purified antibodies.

For the studies described herein, we used recombinant human IDE (rhIDE) that we produced in *E. coli* essentially as described in (26). We expressed both the WT form of the protein and, in the same manner, a E111Q mutated form of the enzyme, in which a point mutation at the catalytic site markedly reduces the enzyme’s catalytic activity (**Figure 1A**) (30). The genes encoding the two forms of IDE were cloned into a pET28a+ plasmid backbone, with a His tag at their C-terminus.

### Affinity Selection (Bio-Panning) of Human Antibody Phage Display Library for Isolating Binders to IDE

The antibody phage display technique was utilized using the “Ronit 1” human synthetic antibody phage display library as previously described (31). Recombinant IDE, WT, and mutated (catalytically inactive E111Q) were obtained as described in *Materials and Methods*. The library was subjected to four affinity-selection cycles on 10 µg WT rhIDE-coated plates for 2 h at RT where WT IDE was used as bait. During two of the cycles, depletion of the library phages on IDE E111Q was carried out prior to positive affinity selection on the WT enzyme, with an intention to isolate antibodies that bind with high affinity to the catalytic site of the WT enzyme. At the end of each affinity-selection cycle, the bound phages were eluted with 100 mM TEA pH = 13 and immediately neutralized with 1M Tris–HCl pH = 7.4. Eluted phages were used to infect XL1-blue *E. coli* and grown to logarithmic phase for clonal amplification. Phage for the next affinity selection cycle were “rescued by” incubation of infected *E. coli* with 10^10^ CFU/ml M13KO7 helper phage overnight at 37°C with shaking at 250 rpm. Virions from bacterial growth supernatant were precipitated in 20% polyethylene glycol (PEG)/NaCl and suspended in phosphate-buffered saline (PBS) before being used in the next affinity-selection (panning) cycle. After the fourth cycle of affinity selection, IDE-specific scFv-displaying phages were identified by monoclonal phage ELISA. Binders were verified for specificity by testing their binding to several control antigens. The three validated binders were reformatted for production as soluble antibodies and tested in three formats: MBP-scFv, human IgG1 produced as “Inclonals,” and reverse-chimeric IgGs produced in mammalian cells culture.

### Expression and Purification of IDE-Specific “Inclonal” Human IgG

The “Inclonals” expression and refolding protocol were carried out as previously reported (32–34).

### Production of Reverses Chimeric Antimouse IDE Antibodies

To convert the antibodies to full-size reverse-chimeric IgGs, they were cloned into pcDNA 3.4 plasmid backbone. These plasmids are based on the CMV promoter-controlled pcDNA3.4 vectors that are provided as the “Antibody Expressing Positive Control Vector” part of the Expi293™ kit for transient transfection-based expression. The kit also provides the Expi293F™ cells (Thermo Fisher, Rockford, IL, USA, #A14635). The cloning antibody variable domains into IgG expression vectors was carried out as previously described (35). Cloning was carried out by PCR amplification of the antibodies heavy and light variable domains followed by cloning into pcDNA 3.4 plasmids already carrying the corresponding constant domains by Gibson assembly (36). Transfection of Expi293F™ cells with the different pcDNA3.4 vectors was performed by ExpiFectamine™ transfection kit (Gibco, #A14524), according to manufacturer recommendations (Life Technologies, Eugene, OR, USA). For each transfection, a total amount of 30 µg plasmid DNA comprised of 3:1 molar ratio of the IgL and IgH, respectively. Transfection, cell growth, and collection of conditioned medium were carried out as recommended by the vendor (Life Technologies Expi293™ kit for transient transfection-based expression). The antibodies-containing conditioned medium was harvested by centrifugation (Sorvall GSA rotor) for 10 min, at 4°C at 8,000 rpm, 6–7 days after transfection. Reverse chimeric mouse IgG1 mAbs were purified on Protein G columns, according to the manufacturer recommendations (GE Healthcare, Chicago, IL, USA). Prior to purification, antibody-containing media were buffered with 20 mM phosphate buffer, pH 7, and filtered. mAbs were eluted in 1 ml fractions, and the pH was neutralized by 0.25 ml of 1.5M Tris–HCl pH 8.8. Buffer exchange to sterile Dulbecco’s PBS (DPBS) (Sigma) was performed on 10-kDa Amicon^®^ Ultra Centrifugal Filters (Millipore Sigma, Burlington, MA, USA) or PD-10 desalting columns (GE Healthcare, Chicago, IL, USA).

The antibodies were collected by centrifugation and concentrated to a final concentration of 1–2 mg/ml using a centrifugal filter concentrator. Purified antibodies were stored at −80°C in small aliquots. Protein concentration was determined by measuring the absorbance of the protein at O.D. 280 nm in the Thermo Scientific NanoDrop™ 2000c spectrophotometer and dividing the absorbance value by the extinction coefficient factor of the protein (extinction coefficient was calculated by http://www.expasy.org/tools/protparam.html).

### Evaluation of Antibodies Binding to IDE by ELISA

Ninety-six‐well ELISA plates (Nunc, Roskilde, Denmark) were coated overnight at 4°C with 2.5–5 µg/ml of rhIDE WT (IDE) or ACE1 or ACE2 or PBS in PBS and non-relevant proteins such as bovine serum albumin (BSA), His trap-purified protein (His), and MBP-LacZ. Following three washes with PBS containing 0.05% Tween-20 (PBST), the wells were blocked for 1 h at room temperature (RT) with 3% w/v skim milk (232100, Difco, Sparks, MD, USA) in PBS. Wells were than incubated for 2 h at RT with IDE-specific antibodies in different formats: as phage displayed scFv clones, with serial dilutions, as MBP-scFv, and as hIgG or as rcIgG in different concentrations. Bound phages were detected with mouse anti-M13 antibody (1 h at RT) followed by incubation with horseradish peroxidase (HRP)-conjugated goat antimouse secondary antibody (115-035-003, Jackson ImmunoResearch Laboratories, West Grove, PA, USA, ME; 1:5,000 in PBS) for 1 h at RT. Human IDE-specific IgG antibodies were incubated with HRP-conjugated goat anti-human (109-035-088, Jackson ImmunoResearch Laboratories, ME; 1:5,000 in PBS) or anti-mouse (115-035-062, Jackson ImmunoResearch Laboratories, ME; 1:5,000 in PBS) for 2 h in RT. Following three washes with PBST, 3,3′,5,5′‐tetramethylbenzidine (TMB; eBioscience, San Diego, CA, USA) was added until color appeared. The reaction was stopped by adding 1M H_2_SO_4_ and analyzed using a microplate ELISA reader at 450 nm.

### Fluorescent Labeling of rcH3-IgG

rcH3-IgG was reconstituted in PBS at pH 7.4 at a concentration of 1 mg/ml. For labeling, the pH of 40 μg of protein solution was adjusted to pH 8.3 using 1M NaHCO_3_ (Merck, Rehovot, Israel) followed by addition of Alexa Fluor 647 NHS ester (Thermo Fisher) at a molar dye-to-protein ratio of 3:1. Samples were incubated at 4°C overnight, and free dye was removed by using a Pierce 1 ml chromatography cartridge desalting (Thermo, USA) with PBS at pH 7.4 as a buffer.

### Affinity Measurements of Binary Equilibrium by Microfluidic Diffusional Sizing

All microfluidic experiments were performed on a Fluidity One-W Serum instrument (Fluidic Analytics, Cambridge, UK) (37). Alexa-647-labeled rcH3-IgG (20 nM) was used, and serial dilution of rhIDE starting at 300 nM was performed. To determine the affinity of IDE binding, Alexa-Fluor-647-labeled rcH3-IgG (20 nM) was mixed with unlabeled IDE at decreasing concentrations (300 nM with 6 additional × 3 dilutions) in PBST and incubated at 4°C for 1 h. To measure complex formation by microfluidic diffusional sizing (MDS), 5 μl of sample was pipetted on a microfluidic chip, and analysis was performed at the 1.5−8.0 nm setting on a Fluidity One-W Serum instrument (center wavelength for excitation is 630 nm with a bandwidth of 38 nm and center wavelength for emission is 694 nm with a bandwidth of 44 nm; Fluidic Analytics, Cambridge, UK). Error bars shown in figures are standard deviations from triplicate measurements. The equilibrium dissociation constant (KD) was determined by nonlinear least-squares (NLSQ) fitting (Prism, GraphPad Software).

### Comparing Binding of H3 IgG to Native IDE Compared to Binding Heat-Denatured IDE by ELISA

This was carried out to understand whether H3 IgG binds to a linear or to a conformational epitope of IDE. A solution of 5 μg/ml rhIDE in PBS was prepared and either used directly to coat half of an ELISA plate or denatured by heating at 80°C for 20 min followed by rapid chilling on ice before using it to coat the other half of the ELISA plate. The plate was coated overnight at 4°C with 50 μl/well of either native or denatured rhIDE. On the following day, the plate was washed once with 300 μl/well of PBST and was blocked with 300 μl/well of 3% skim-milk solution in PBS for 1 h at 37°C. Next, the plate was washed three times with 300 μl/well of PBST, and rcH3-IgG at concentrations of 100, 50, 25, 12.5, 6.25, 3.125, and 1.625 nM was applied to the wells of three columns of wells coated with native or with heat-denatured rhIDE. This was followed by washing these wells three times with 300 μl/well of PBST/well followed by adding 50 μl/well of HRP-conjugated goat antihuman IgG diluted × 5,000 in PBST. The plate was left for 1 h at room temperature and then washed three times with 300 μl/well of PBST. Finally, 50 μl/well of the HRP substrate TMB was added until color appeared. The reaction was stopped after 2 min by adding 50 μl/well 1M H_2_SO_4_ and analyzed using a BioTek microplate ELISA reader. The optical density was measured at 450-nm wavelength. The results are presented as mean ± SEM (n = 3). To quantify total rhIDE coated onto the wells, the other half of the plate was incubated with HRP-conjugated goat anti-His-tag antibody, diluted 2,000×, 4,000×, 8,000×, 16,000×, 16,000×, 32,000×, 64,000×, and 128,000× in PBST. The plate was left for 1 h at room temperature and then washed three times with 300 μl/well of PBST. Finally, 50 μl/well of the HRP substrate TMB were added until color appeared. The reaction was stopped after 2 min by adding 50 μl/well 1M H_2_SO_4_ and analyzed using a microplate ELISA reader. The optical density was measured at 450-nm wavelength. The results are presented as mean ± SEM (n = 3).

### IDE Insulin Digestion Assay

Various concentrations of IDE inhibitors were incubated with 1.5 µg/ml rhIDE for 1/2 h at RT. Next, recombinant human insulin (41-975-100, Biological Industries, Kibbutz Beit Haemek, Israel) diluted in Mercodia ultrasensitive mouse insulin ELISA (10-1249-01) calibrator 0 was added to the tubes and incubated for 1 h at 37°C. From each tube, 25 µl were taken and evaluated for residual insulin concentration using the Mercodia ultrasensitive Mouse Insulin ELISA kit. Absorbance at 450 nm was recorded by a Spectrafluor plus microplate reader (Tecan, Männedorf, Switzerland).

### Mice

Male C57BL/6 mice were purchased from the Jackson Laboratory; IDE KO mice were kindly received from Prof. Dennis Selkoe’s laboratory (38). All experiments were in accordance with Tel Aviv University guidelines and approved by the Tel Aviv University (TAU) animal care committee for animal research.

### Streptozotocin Diabetes Mouse Model

Ten-weeks-old C57BL/6 mice were fasted overnight, after which they were intraperitoneally injected with 150 mg/kg of STZ diluted in 100 nM citrate buffer at pH =4.5. All mice developed hyperglycemia within 2 days from the STZ injection. At day 3 after STZ injection, each mouse was intraperitoneally injected with 10 mg/kg of reverse chimeric mAbs.

### Oral Glucose Tolerance Test

Mice were fasted for 2 h after which they received a single i.p. injection of reverse chimeric mAbs (10 mg/kg), followed by additional 4 h of fasting (six in total). At the end of this time period, mice were given glucose orally, using a gavage needle, at a dose of 2 g/kg. Blood glucose was measured prior to glucose administration (time 0) and 15, 30, 60, 90, and 120 min after glucose administration using the Contour blood glucose meter (Bayer, Elkhart, IN, USA).

### Testing the Binding Specificity of H3 IgG to Tissue-Expressed IDE by ELISA

C57BL/6 J WT and IDE−/− mice were euthanized under CO_2_ and were transcardially perfused with 20 ml ice cold PBS. Mice were then quickly dissected to collect the liver, kidney, and spleen. The isolated organs were cut into small pieces using razor blades on a chilled glass stage and were collected into ice-cold IDE extraction buffer. Tissue was homogenized in extraction buffer using mechanical pestle to obtain a clear lysate. The lysate was left on ice for 30 min and was later centrifuged at 13,000 rpm for 15 min at 4°C. The supernatant was used for subsequent ELISA. The ELISA for quantifying IDE in the cell extracts was carried out as described before for measuring IDE levels in sera (26).

### Viability Assay of Stress-Induced HepG2 Cell Line

Human hepatocellular carcinoma cells (HepG2) were seeded, 2.5 × 10^4^ cells/well in 96-well plate, containing Dulbecco’s modified Eagle’s medium with 1 g/L D-glucose (low glucose), at 37°C, 5% CO_2_ for 6 h and later treated with 5 µM Rotenone at 37°C, 5% CO_2_ for 4 h. Then, the medium containing Rotenone was replaced by fresh medium, and the plate was kept at 37°C, 5% CO_2_ overnight. Next, the cells were treated with or without different antibodies (as indicated) at a final concentration of 1 µM for 22 h. After incubation, 50 µl/well XTT reagent (Biological industries, Israel) was added according to suppliers’ recommendations at 37°C, 5% CO_2_ for 2 h. Plate was analyzed with an EMax Plus Microplate Reader at 450 and 630 nm. At all stages, volume was 100 µl/well unless mentioned otherwise.

### Insulin Tolerance Test

Mice were fasted for 2 h after which they received a single i.p. injection of reverse chimeric mAbs (10 mg/kg), followed by additional 4 h of fasting (6 h in total). At the end of this time period, the mice were given an i.p. injection of insulin (0.5 U/kg, dissolved in PBS). Blood glucose was measured prior to glucose administration (time 0) and 15, 30, 60, and 90 min after glucose administration. The experiment was repeated 11 days later (with no further injection of antibody). In addition to measuring blood glucose levels, we measured blood insulin concentration at 90 min following insulin injection. Blood glucose levels were measured using the Contour blood glucose meter (Bayer, Elkhart, IN, USA); serum insulin levels were measured using the Mercodia ultrasensitive Mouse Insulin ELISA kit as described above.

### Serum Collection

Following the ITT and the injection of reverse chimeric mAbs, mice were bled every 3 days from the facial vein using, a 27-G needle. Blood samples (no more than 120 µl each time) were put in 1.5-ml Eppendorf tubes at RT for 1 h. The blood clots were removed from the tubes using a needle, and the tubes were put on ice for 40 min. Next, the tubes were centrifuged for 10 min at 4°C at 2,300 rpm; the clear supernatant was then taken to a new tube for a further cleaning cycle. The supernatant was taken into a new tube and kept at −20°C.

### Detection of Reverse-Chimeric Antibodies in Serum Samples

Ninety-six‐well ELISA plates were coated over night with 50 μl/well of 5 μg/ml of IDE in PBS at 4°C. Following three washes with 300 μl/well of PBST, wells were blocked with 300 μl/well of 5% (w/v) skim milk in PBS, for 1 h in RT. For the detection of the IDE-specific antibody (rcH3-IgG), serum samples were diluted 1:2,700 in PBS, whereas control antibody-injected samples served as a control. Known concentrations of the rcH3-IgG antibody diluted in a 1:2,700-diluted sample of control mice served as a reference for the measurement of IDE concentrations. Samples were incubated for 2 h at RT. Following three washes, wells were incubated with 50 μl/well of HRP-conjugated goat anti-mouse antibody (1:5,000 in PBS) for 1 h at RT. Following three washes with PBST, 50μl/well of TMB was added until color appeared. The reaction was stopped by adding 50 μl/well of 1 M H_2_SO_4_ and analyzed using an ELISA reader at 450 nm.

### Statistical Analysis

GraphPad software (GraphPad Prism v8) was utilized for all the statistical analyses. Data comparisons were carried out using either an unpaired two-tailed Student’s t-test when two groups were compared or one-way ANOVA (with Bonferroni’s *post-hoc* test) when three or more groups were analyzed. Each experiment was repeated at least three times. p < 0.05 was considered significant.

## Results

### Expression and Purification of WT and Mutated IDE Proteins

For the studies described herein, we used the recombinant human IDE that we produced in *E. coli* as described in (26). The purified rhIDE was used as the antigen for both the screening and for immunization. We expressed both the WT form of the protein and in the same manner a E111Q-mutated form of the enzyme, in which a point mutation at the catalytic site markedly reduces the enzyme’s catalytic activity (30, 39).

### Isolation and Characterization of IDE-Specific Antibodies

**Figure 2:**
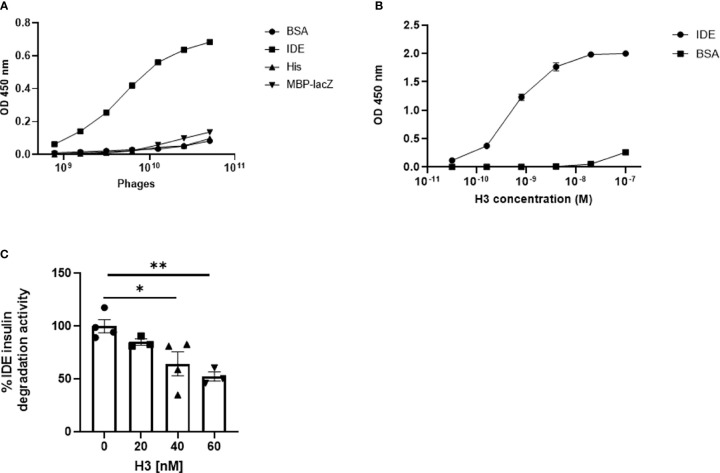
Binding of IDE by IDE-specific phage displayed antibodies and binding and inhibition of IDE by “Inclonal” human IgGs. **(A)** Analysis of IDE binding by H3 scFv displaying phages in ELISA. The analyzed scFv displaying phages were added in serial dilutions to ELISA plate wells previously coated with 2.5 µg/ml of rhIDE WT (IDE) and non-relevant proteins: BSA, a His trap-purified protein (His) and MBP-LacZ. Bound phages were detected with mouse anti-M13 antibody followed by HRP-conjugated goat anti-mouse secondary antibody. The results are presented as mean ± SEM (n = 3). **(B)** Analysis of IDE binding by purified “Inclonal” human IgG in ELISA. The analyzed antibodies were added in serial dilutions to ELISA plate wells coated with 2 µg/ml of IDE WT and BSA. Bound antibodies were detected with a HRP-conjugated goat anti-human secondary antibody. The results are presented as mean ± SEM (n = 4). **(C)** Evaluation of inhibition of IDE-mediated cleavage of insulin by human H3 IgG by ELISA. IDE (1.2 µg/ml) was incubated with 20, 40, or 60 nM of the antibodies 1/2 h at 25°C, followed by incubation of 1.5 µg/L of human insulin 1 h at 37°C. Residual insulin was later analyzed using Mercodia ultrasensitive mouse insulin ELISA kit. The results are presented as mean ± SEM (n = 3–4). *p < 0.02. *p = 0.0227, **p = 0.0075.

The purified rhIDE along with the mutated IDE was used as bait in order to isolate specific scFv clones that recognize specific epitopes of WT IDE. Antibody phage display was utilized using the “Ronit 1” human synthetic antibody phage display library (31) as described in *Materials and Methods*. After four cycles of affinity selection on WT IDE, we identified three IDE-specific scFv-displaying phage clones (two of which are not presented herein), one of which, later proven to be an IDE-neutralizing antibody, was named H3 (see **Figure 1B**). As shown in **Figure 2**, serial dilutions of phages displaying the H3 (**Figure 2A**) bind IDE well and show little to no binding to BSA, a His trap-purified recombinant fusion protein (His), and MBP-LacZ proteins that were used as negative controls.

For initial evaluation of full-size IgGs, we first produced the antibodies as “Inclonals”; IgGs expressed in an *E. coli* expression system (32). The isolated “Inclonal” antibody showed a marked binding to IDE and an EC_50_ of about 1 nM (**Figure 2B**). The antibodies showed very little binding to BSA (negative specificity control).

We further examined the ability of human H3 IgG to inhibit IDE activity. We found that it inhibits insulin digestion by IDE in a dose-dependent manner (**Figure 2C**).

**Figure 3:**
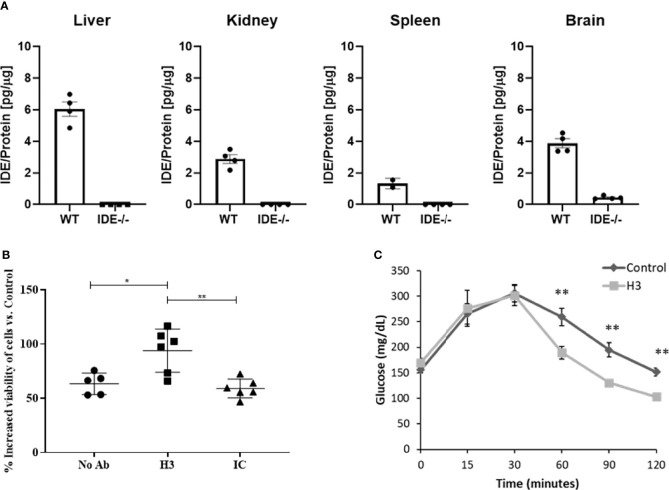
Evaluation of binding specificity of human H3 IgG to tissue-expressed IDE and its activity in cell culture and in mice. **(A)** IDE levels in the liver, kidney, spleen, and brain protein extracts of WT and IDE−/− mice were measured by ELISA. There was no detectable quantity of IDE from any organ in IDE−/− mice. The results are presented as mean ± SEM (n = 4). **(B)** Anti-IDE human H3 IgG protects HepG2 cells from oxidative stress. HepG2 were treated with 5 μM Rotenone for 4 h. Then, media were discarded, and cells were left to recover overnight with 1 μM antibody treatment as indicated. Twenty-four hours later, cell viability was determined by XTT-based cell proliferation kit. The results are presented as mean ± SD (One way ANOVA; *p = 0.0078; **p = 0.002). **(C)** Thirty minutes before oGTT, 6-month-old male mice received a single i.p. injection of scFv at concentration of 2 mg/ml (20 mg/kg) or PBS (control). Blood glucose was measured before glucose administration (0) and 15, 30, 60, 90, and 120 min after. The results are presented as mean ± SEM (n = 5). **p < 0.01. One-way ANOVA with Bonferroni correction was performed.

We assessed the binding specificity to human H3 IgG to IDE by assessing its ability to recognize IDE from tissue extracts obtained from WT vs. IDE−/− mice, using ELISA as described before (26). As shown in **Figure 3A**, the antibody did not show binding to proteins extracted from IDE−/− mouse tissues but did bind to proteins extracted from WT mouse tissues. Together, these results suggest that the human H3 IgG binds IDE with high affinity and specificity.

While we expected that H3 IgG will affect only the secreted IDE levels, we assessed potential toxicity to liver cells, which were reported (40) (and as shown in **Figure 3A**) to express high IDE levels. We tested the hepatocyte cell line HepG2 (40). Herein, cells were subjected to oxidative stress using rotenone, which is known to increase oxidative stress, which may lead to IDE elevation (41). Cells were treated with 5 μM rotenone for 4 h. Then, media were discarded, and cells were left to recover overnight with human H3 IgG, isotype control, or left untreated, and cell viability was determined and normalized to untreated cells. We could not detect any toxicity associated with human H3 IgG treatment and, as shown in **Figure 3B**, even found a significant increase by ~50% in the survival of cells treated with human H3 IgG compared to the controls. We further tested whether treatment of WT mice with H3 single-chain Fv (scFv) could reduce IDE activity, leading to higher insulin activity and reduced glucose clearance in oGTT. C57/BL6 male mice at the age of 6 months were treated with either H3 scFv (20 mg/kg, n = 5) or PBS (control, n = 5) 30 min prior to glucose administration. Blood glucose was measured prior to oral administration of glucose (0) and 15, 30, 60, 90, and 120 min after. At time intervals 60, 90, and 120, the blood glucose levels of scFv H3-treated mice were significantly lower than the control group and PBS-treated mice (**Figure 3C**).

### Conversion of the IDE-Specific Antibodies to Reverse-Chimeric IgGs

**Figure 4:**
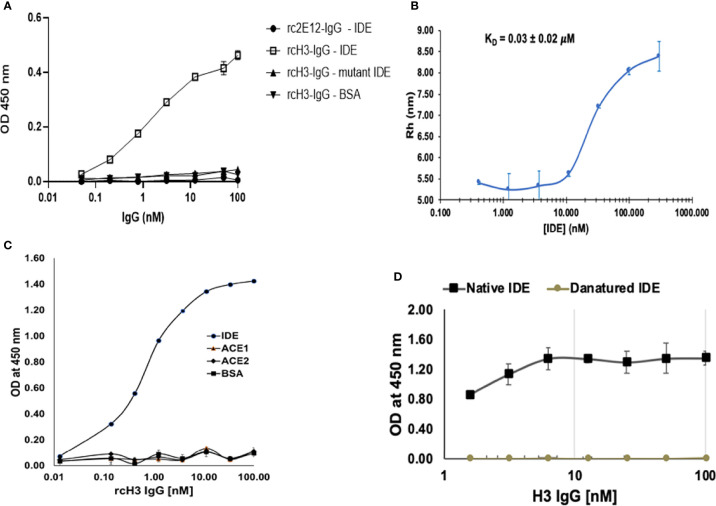
Reverse chimeric H3 IgG binds IDE specifically and in a conformation-dependent manner. **(A)** Evaluation of binding to WT and to mutant IDE by reverse-chimeric H3 and by the isotype control, reverse-chimeric 2E12 IgGs by ELISA. The results are presented as mean ± SEM (n = 3). **(B)** Binding affinity of rcH3 to rhIDE measured by MDS. A binding experiment of rhIDE to Alexa-647-labeled rcH3-IgG using diffusional sizing (MDS) (37). Bars show the average of triplicate measurements with the error bars representing the standard deviation. From the fit, the dissociation constant KD = 30 nM could be calculated. **(C)** Specificity of reverse-chimeric H3 to IDE vs. ACE1 and ACE2 was evaluated by ELISA. **(D)** rcH3-IgG recognizes a conformational epitope on rhIDE. rcH3-IgG at concentrations of 100, 50, 25, 12.5, 6.25, 3.125, and 1.625 nM was applied to the wells of three columns of wells coated with native or with heat-denatured rhIDE. The ELISA was carried out as described in *Materials and Methods*.

Chimeric antibodies were a critical milestone in the history of therapeutic antibody development. Chimeric antibodies are recombinant IgGs where the variable domains are from a mouse antibody, while the constant domains are human sequences. Compared to mouse monoclonal antibodies (mAbs), chimeric antibodies are far less immunogenic, limiting the elicitation of human-anti-mouse antibodies upon administration to human patients (42). Conversely, reverse-chimeric antibodies are antibodies with human variable domains and murine constant domains (43). In addition to being useful for antibody discovery in transgenic mice, reverse-chimeric antibodies can be very useful for treating mouse models, to avoid eliciting a mouse-anti-human immune response. In order to evaluate the efficacy of our IDE-specific antibody in mouse models, we converted it to a reverse-chimeric IgG (rcIgG) (mouse IgG1 isotype). The rcH3-IgG was purified as described in *Materials and Methods* to a high level of purity (**Supplementary Figure S1A**). It was tested for binding to IDE, BSA, and the E111Q IDE mutant (**Figure 4A**). Antibody rc2E12 that does not bind IDE served as an isotype control. As shown in **Figure 4A**, rcH3-IgG binds IDE with high affinity (EC_50_ of 1.62 nM), similar to the human H3 Inclonal reported above. In addition to ELISA, we accurately measured the KD of rhIDE to rcH3-IgG by MDS (**Figures 4B**). As the IDE concentration was decreased, less of the fluorescently labeled rcH3-IgG was found in the protein complex, and the effective hydrodynamic radius (Rh) decreased accordingly. Based on a 1:1 equilibrium binding model, the KD was determined to be 30 nM, similar to values that we obtained by ELISA (see **Figure 4A**). It was reported that other IDE inhibitors may bind to the zinc in its catalytic site and may interfere with other important similar zinc metalloprotease enzymes, such as angiotensin-converting enzyme (ACE) (44). As presented in **Figure 4C**, the rcH3-IgG showed specificity to IDE and did not bind to other zinc metalloproteinase such as ACE1 or ACE2.

In order to define whether rcH3-IgG recognized the conformation of active rhIDE, we compared the difference in binding to native compared to heat-denatured rhIDE. As shown in **Figure 4D**, thermal denaturation of rhIDE resulted in a very significant reduction in the ELISA signal resulting from detection with rcH3-IgG (down to almost negligible signal). We also evaluated how heat denaturation affected coating of the ELISA plate compared to coating with native IDE in the same plate. As shown in **Supplementary Figure 1B**, we found that thermal denaturation of rhIDE resulted in lowering (but not elimination) of the ELISA signal where rhIDE was detected using serial 2× dilutions of an anti-His-tag antibody (that recognizes a linear epitope), suggesting that denatured rhIDE might also bind less efficiently to the wells of the ELISA plate. Combined, these results suggest that H3 IgG recognizes specifically a conformational epitope of active rhIDE.

### IDE-Specific Antibodies Improve Insulin Signaling in a Diabetes Mouse Model

**Figure 5:**
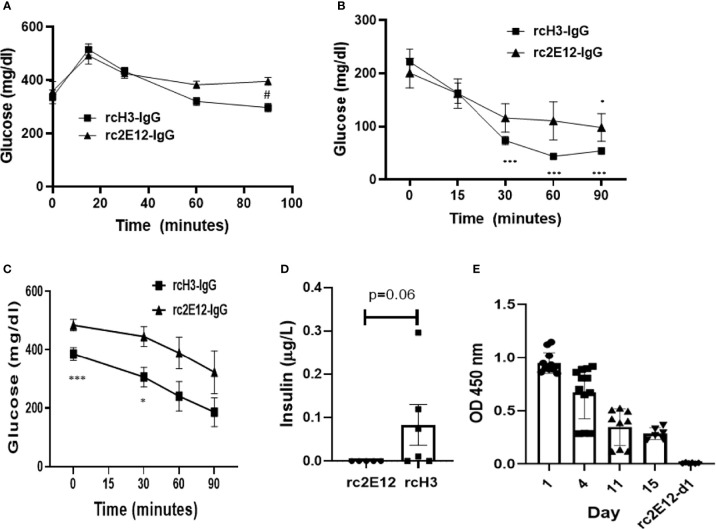
The reverse-chimeric IDE-specific antibody H3 increases blood insulin levels and reduces blood glucose levels in STZ-induced diabetic mice. **(A)** Two days after STZ injection, mice were administrated i.p. with IDE-specific antibody or control antibody and were tested by **(A)** oGTT assay (n = 5–6 mice in each group, ^#^p < 0.01 *between* groups) or **(B)** ITT assay (n = 5 mice in each group, *p < 0.05, ***p < 0.001 *within* group). **(C)** ITT assay following 11 days of antibody administration in STZ-treated mice (n = 5–6 mice in each group, *p = 0.015, ***p = 0.008). **(D)** Insulin serum levels following 11 days of antibody administration in STZ-treated mice assessed by Mercodia ultrasensitive mouse insulin ELISA. The results are presented as mean ± SEM. **(E)** Levels of IDE-specific antibodies at different days following administration (n = 2–4 mice in each group). The results are presented as mean ± SEM.

It was previously suggested that inhibition of IDE may improve insulin activity in a diabetes mouse model (22). We further assessed the ability of rcH3-IgG to reduce glucose levels (oGTT) and improve insulin activity (iTT) in STZ-treated mice. We administered i.p. either rcH3-IgG or an isotype control antibody to STZ-treated mice, 1 h prior to testing oGTT and iTT. Following a glucose challenge (oGTT), both control and rcH3-IgG-treated mice exhibited a rise in blood glucose levels. However, rcH3-IgG-treated mice exhibited lower levels of glucose through time, compared to the isotype control-treated mice (p < 0.05). *Post-hoc* analysis revealed that rcH3-IgG-treated mice had significantly lower glucose levels 90 min after glucose administration compared to the isotype control-treated mice (p < 0.05; **Figure 5A**). These results suggest that treatment with rcH3-IgG improves glucose levels in a diabetes mouse model. We further assessed the ability of rcH3-IgG to improve insulin activity. We found that while the rcH3-IgG-treated mice exhibited a significant reduction in glucose levels starting from 30 min after the administration of insulin (p < 0.001), the isotype control-treated mice exhibited a significant reduction in glucose levels only after 90 min (p < 0.05; **Figure 5B**). To assess the long-term effect of rcH3-IgG, we measured glucose 11 days after antibody administration (without additional antibody administration) and found that the blood glucose levels were still significantly lower in the rcH3-IgG-treated mice as compared to the rc2E12-IgG-treated mice (**Figure 5C**). Furthermore, the rcH3-IgG-treated mice showed significantly improved iTT test results (**Figure 5C**). In addition, at this point, we measured serum insulin levels and found that they were higher in the rcH3-IgG-treated mice as compared to the rc2E12-IgG-treated mice (**Figure 5D**). Indeed, we tested the half-life of the rcH3-IgG antibody in the sera of the treated mice and found that it was about 11 days (**Figure 5E**).

## Discussion

During our studies focused on IDE, we developed IDE-specific antibody (H3) that inhibits IDE activity. Furthermore, we showed its potential to improve insulin activity in a diabetes mouse model.

IDE is a metalloprotease involved in insulin degradation, and mice and human IDE share 95% amino acid identity with an identical catalytic site. Currently, the use of small molecule inhibitors of IDE is limited in clinical trial due to their non-specificity to other zinc metalloproteinase (45). It has been previously suggested that IDE activity is linked to its conformational stage, and changes within the structure may affect its activity (45). We applied a unique approach for isolating antibodies that may affect the enzyme activity. The isolation process of the IDE-specific scFv-displaying phages was subjected to depletion of rhIDE E111Q binders before positive selection for WT IDE binders. The difference between the two rhIDE isoforms is one amino acid in the catalytic site that renders the enzyme catalytically compromised (46). The depletion step probably caused a loss of IDE-specific clones that bind remotely from the catalytic site and improved the chances of recovering antibodies that bind vicinal to the catalytic site. Furthermore, the antibody could detect WT IDE in its native conformation but failed to recognize mutant IDE (**Figure 4A**) or heat-denatured IDE (**Figure 4D**), suggesting that its epitope is conformational.

IDE is an essential enzyme for insulin activity. Indeed, IDE knockout causes hyperinsulinemia and hyperglycemia in mice (38). However, this may be due to its cellular role in monitoring the release of insulin from its receptors (47). Furthermore, partial IDE depletion in IDE-heterozygote mice did not lead to any reported pathology. IDE was shown to regulate the levels of serum insulin (38), and polymorphisms in the *IDE* gene were linked with T2D in rats (48) and humans (49). Recently, we reported an increase in serum IDE concentration in metabolic syndrome patients who are at high risk of developing diabetes (26) Furthermore, inhibition of IDE activity as potential treatment for diabetes was previously suggested (22) and show efficacy without aberrant toxicity (25). H3 antibody shows high specificity to IDE and does not recognize any other tissue proteins isolated from selected tissue such as the liver, kidney, spleen, and brain. Furthermore, while other reports suggest that impairment in IDE activity in liver cells can lead to stress and impaired activity (50), here, we showed that treatment with H3 antibody following oxidative stress in hepatic cell line increase survival. Those results may suggest that targeting the secreted form of IDE by an antibody may prevent interference with essential intercellular IDE activity (50). Of note, H3 antibody did not recognize other essential zinc metalloproteinase such as ACE1 or ACE2, suggesting both its specificity and potential safety.

Due to their large size, unless they bind to a cell-surface internalizing target, antibodies do not penetrate cells. Therefore, under pathological conditions of hypoinsulinemia in diabetes, antibodies inhibiting the secreted form of IDE should increase insulin levels and ameliorate pathological features of the disease. Indeed, we could show that administration of an IDE-specific antibody can improve insulin activity in an environment where there are limited insulin levels such as in STZ-treated mice. We also show that H3 MBP-scFv has efficacy in WT mice. In addition, we could detect the rcIgGs up to 11 days following administration, which is in the normal range for murine IgGs in mice (51). Of note, as shown in iTT assay (without additional antibody administration) and by measurement of serum insulin levels at day 11, we found that the glucose levels were lower and insulin levels were higher in rcH3-treated mice compared to rc2E12 (isotype control)-treated mice.

Of note, while small molecules may interfere with intracellular IDE activity, antibodies targeting the cell surface or secreted forms of IDE do not affect the intracellular IDE pool directly, which may be more beneficial for controlling insulin levels. Another advantage may be linked to a reduced clearing of antibodies from the circulation as compared to small molecules. Blocking IDE activity may be beneficial for the maintenance of high circulating insulin levels and other substrates of IDE in pathological conditions such as diabetes. In pathological stages that require exogenous insulin injection, IDE inhibition may serve as a therapeutic intervention, as it will decrease the necessary amounts of administrated insulin and will increase its effective half-life in the serum. Beneficial insulin properties are not limited to glucose regulation but also to the promotion of beta-cell regeneration. Neutralizing antibodies can inhibit IDE-dependent insulin degradation by either directly binding and blocking the active site of IDE or by binding to a site on IDE that affect conformational changes or dimerization. Of note, the H3 antibody preferentially recognizes only the native form of IDE, which suggests that its affect may be linked to the altering active conformational form of the enzyme. Further research needs to be done in order to identify such interactions.

It was suggested that besides IDE, cathepsin may play an important role in the degradation of intracellular insulin (52). Nevertheless, it was reported that fasting serum insulin levels in IDE-KO is higher than those in WT mice (53). While different IDE inhibitors show efficacy in modulating glucose levels, IDE’s exact role in glucose metabolism might be complex and needs further investigation (39).

In conclusion, we present here a new approach that may be potentially used to treat diabetes using an antibody targeting IDE. This approach may have limited potential to toxicity due to its high specificity to IDE. Further research using our approach will increase our understanding regarding the role of IDE in diabetes and may provide novel therapeutic approaches in diabetes and related complications.

## Data Availability Statement

The original contributions presented in the study are included in the article/**Supplementary Material**. Further inquiries can be directed to the corresponding authors.

## Ethics Statement

The animal study was reviewed and approved by Tel Aviv University guidelines and approved by the TAU animal care committee for animal research.

## Author Contributions

OF, ML, YN, LN, DI, VM, GG, TGR, IB, and DF designed the research and analyzed data. OF, ML YN, DI, VM, TGR, GG, and LN performed the experiments and collected the data. ML, YN, IB, and DF wrote the manuscript. All authors contributed to the article and approved the submitted version.

## Funding

The work was supported by Brainboost grant from Sagol School of Neuroscience (DF) grant from Israel Innovation Authority (DF and IB).

## Conflict of Interest

The authors declare that the research was conducted in the absence of any commercial or financial relationships that could be construed as a potential conflict of interest.

## Publisher’s Note

All claims expressed in this article are solely those of the authors and do not necessarily represent those of their affiliated organizations, or those of the publisher, the editors and the reviewers. Any product that may be evaluated in this article, or claim that may be made by its manufacturer, is not guaranteed or endorsed by the publisher.

## Abbreviations

DM, diabetes mellitus; IDE, insulin-degrading enzyme; rhIDE, recombinant human IDE; mAb, monoclonal antibody; IP, intraperitoneal; MBP, *E. coli* maltose-binding protein; oGTT, oral glucose tolerance test; ITT, insulin tolerance test; rcIgG, reverse-chimeric IgG (with human variable and mouse constant immunoglobulin domains); RT, room temperature (about 25°C); STZ, streptozotocin; T1D, type 1 diabetes; T2D, type 2 diabetes.
