# Supplementary figures and images for: Antibody-Mediated Inhibition of Insulin-Degrading Enzyme Improves Insulin Activity in a Diabetic Mouse Model
Source: Front Immunol. 2022 Mar 8;13:835774. doi: 10.3389/fimmu.2022.835774 (PMC8958001; doi:10.3389/fimmu.2022.835774)

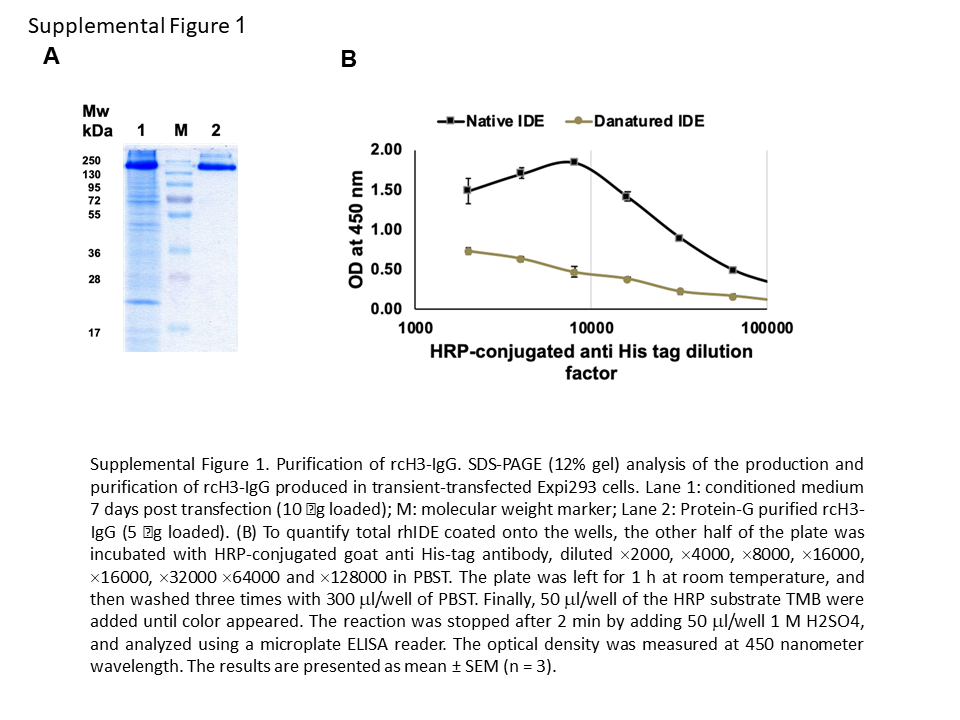

Supplement: Supplementary file 1 [file Image_1.tif]
